# Association between polymorphism of TGFA Taq I and cleft Lip and/or palate: a meta-analysis

**DOI:** 10.1186/1472-6831-14-88

**Published:** 2014-07-11

**Authors:** Cuijuan Feng, Enjiao Zhang, Weiyi Duan, Zhongfei Xu, Yang Zhang, Li Lu

**Affiliations:** 1 Department of Orthodontics, School of Stomatology, China Medical University, No.117 North Nanjing Street, Shenyang 110002, PR China; 2 Department of Oral and Maxillofacial Surgery, School of Stomatology, China Medical University, Shenyang 110002, PR China

**Keywords:** Cleft Lip and Palate, Clip lip, Clip palate, Transforming growth factor alpha, Single nucleotide polymorphism, Meta-analysis

## Abstract

**Background:**

Cleft lip and palate (CL/P) is one of the most common malformations in humans. Transforming growth factor alpha (TGFA) is a well characterized mammalian growth factor which might contribute to the development of CL/P. This meta-analysis aimed to summarize the association between the TGFA Taq I polymorphisms and CL/P.

**Methods:**

We retrieved the relevant articles from PubMed, EMBASE, ISI Web of Science and SCOPUS databases. Studies were selected using specific inclusion and exclusion criteria. The odds ratios (ORs) and their 95% confidence intervals (95% CIs) were calculated to assess the association between TGFA Taq I polymorphism and CL/P risk. Meta-analyses were performed on the total data set and separately for the major ethnic groups, disease type and source of control. All analyses were performed using the Stata software.

**Results:**

Twenty articles were included in the present analysis. There is a significant association between the TGFA Taq I polymorphism and CL/P (C1C2 vs C1C1: OR = 1.67, 95% CI = 1.23-2.25, C2C2 + C1C2 vs C1C1C1: OR = 1.52, 95% CI = 1.15-2.01; C2 vs C1:OR = 1.41, 95% CI = 1.12-1.78). Stratified analyses suggested that the TGFA Taq I polymorphism was significantly associated with CL/P in Caucasians (C1C2 vs C1C1: OR = 1.95, 95% CI = 1.34-2.86; C2C2 + C1C2 vs C1C1: OR = 1.68, 95% CI = 1.18-2.38; C2 vs V1: OR = 1.52, 95% CI = 1.14 -2.02).

**Conclusion:**

TGFA Taq I polymorphism may be associated with the risk of CL/P.

## Background

Facial clefting is one of the most common malformations in humans. Significant differences between populations in the prevalence of cleft lip or palate (CL/P) have been reported, with higher rates found in Asians and American Indians than those observed in Caucasians and Africans. Palate formation is complex, and there are numerous potential untoward possibilities, the most common being delayed shelf horizontalization and inadequate shelf growth [1].

Epidemiologic studies suggest that a number of environmental factors have been examined as risk factors for CL/P, including maternal smoking, exposure to antiepileptic drugs, antiemetic agents and vitamin use during the periconceptual period, maternal metabolic factors, alcohol consumption and exposure to agricultural chemicals [2]. Several studies have suggested that maternal cigarette smoking increased the risk of delivering infants with orofacial clefts [3-6]. It has previously been shown that maternal periconceptional intake of multivitamins containing folic acid decreased the occurrence of CL/P [6-8]. However, there is a study showing the different results [9]. A case–control study showed that CL/P was associated with maternal alcohol consumption [10]. However, Christensen and colleagues found that before the pregnancy there were fewer case mothers drinking alcohol than control mothers [11].

The epidemiologic characteristics and risk factors of CL/P are not clear. There is also a strong genetic component to oral clefts. The host susceptibility factors may play an important role in the development of CL/P. Ardinger and colleagues performed the first study to use a case–control design to test candidate genes [12]. They found a significantly statistical association between CL/P and two of 12 markers in five genes, with an intronic *Taq1* marker in the transforming growth factor alpha (TGFA) gene showing the strongest association. TGFA encoded by a gene mapped at 2p13, is a secretion protein that binds to the epidermal growth factor receptor (EGFR) and is situated at the palate epithelium during palate closing [13]. TGFA may function as a normal embryonic version of EGF-related growth factor [14]. EGF/TGFA and glucocorticoids are believed to regulate the proliferation and differentiation of palatal epithelial cells both *in vitro* and *in vivo*. Moreover, the continued presence of EGF inhibits the fusion process; TGFA is likely to have similar effects. These biological studies suggest that mutations in the TGFA gene might contribute to the development of CL/P, especially for those mutations that affect the timing of the tissue-specific expression of this gene.

The TGFA gene shows a restriction fragment length polymorphism when treated with Taq I restriction enzyme. The mutant allele shows a four-base (TAAT) deletion. In this case, it shows a 178-base pair (bp) C1 allele and a 174-bp C2 allele [15]. TGFA Taq I polymorphism is located at intron 5 and has 602 bp in the 59 direction of the acceptor site of exon 6 [16]. For this polymorphism, C1C1 is wild genotype, C1C2 is heterozygote genotype, and C2C2 is homozygote mutation genotype. In most studies, there are different forms of comparisons such as heterozygote comparison (C1C2 vs. C1C1), homozygote comparison (C2C2 vs C1C1), dominant model (C1C2 + C2C2 vs C1C1), recessive model (C2C2 vs C1C2 + C1C1) and allelic model (C2 vs C1). Ardinger and colleagues first reported association between the Taq I polymorphisms at the TGFA locus and CL/P susceptibility in a case–control study [12]. This finding has since been replicated in some studies [6,15,17-23]. However, there are still controversies of the effect of TGFA polymorphism on the predisposition of this malformation [24-35].

The above inconsistent conclusions in the findings of the studies may be attributed to the size of the samples, the ethnic of the sample population and other reasons. In order to contribute to a better understanding of the role of this gene in the occurrence of cleft lip, cleft lip, or cleft lip and palate, we perform an updated meta-analysis on all available case–control studies, combining data together to reach a larger sample size, to get more statistical power to evaluate the association between CL/P susceptibility and TGFA Taq I polymorphism. Understanding the genetic background and etiology of CL/P is essential for both the risk assessment and findings of effective methods for prevention and treatment.

## Methods

### Data sources

We retrieved the articles using the following terms “Transforming growth factor alpha or TGFA” and “cleft lip or cleft palate or cleft lip and palate” from PubMed, Embase, ISI Web of Science and SCOPUS (Last search was updated on October 2013). There was no any language restriction and the age of participants was not considered as selection criteria. We evaluated potentially relevant publications by examining their titles and abstracts and all studies matching the eligible criteria were retrieved.

### Study selection and data extraction

Eligible studies were selected according to the following explicit inclusion criteria: (a) evaluation of the TGFA Taq I polymorphism and CL/P risks, (b) using the methodology of a case–control study to keep the homogeneity between included studies in the meta-analysis.

**Figure 1:**
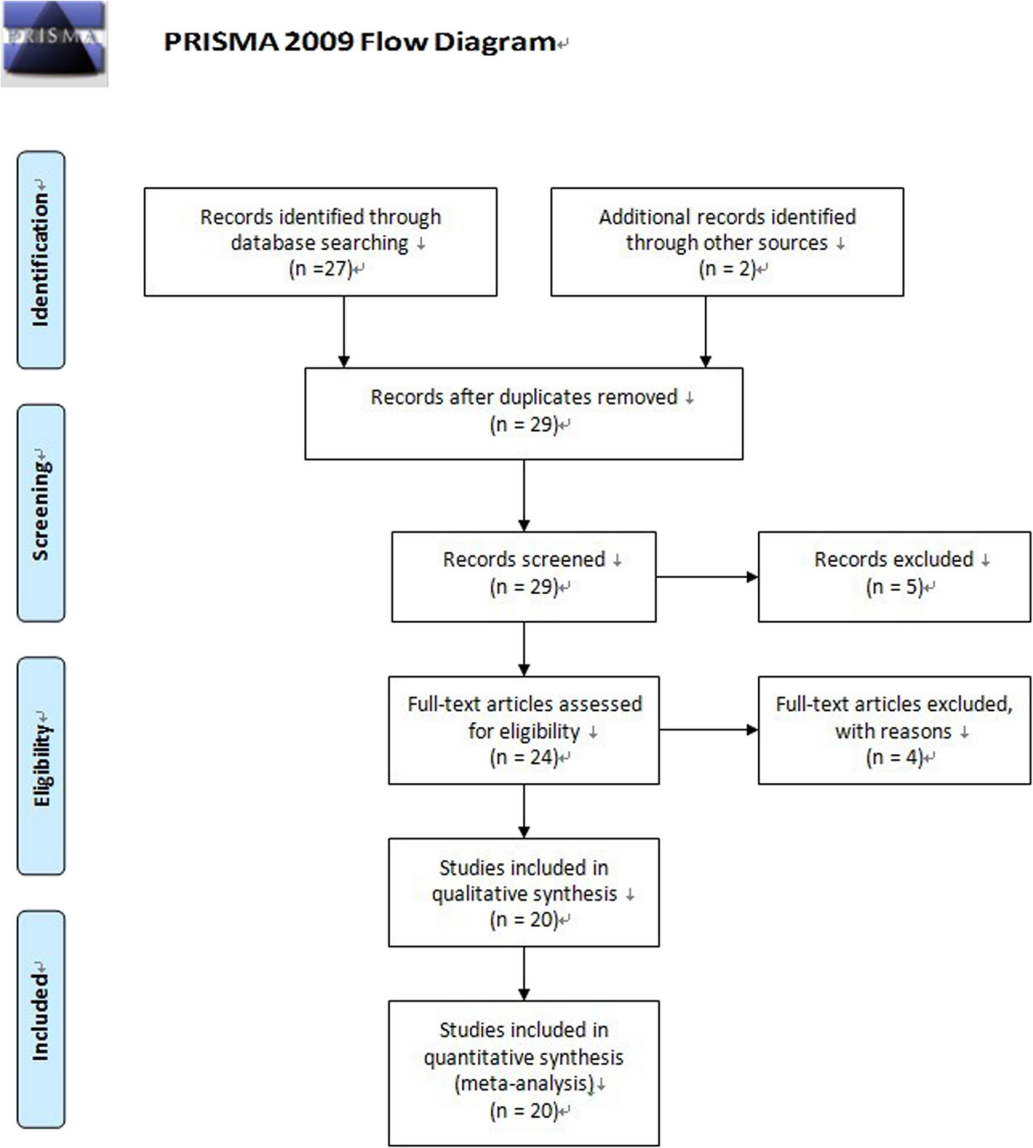
Flow chart of the study selection process.

Duplicate and obviously unrelated articles were eliminated by a single author (C.F.). Abstracts of the remaining articles were examined independently by two authors (C.F. and E.Z.) to determine whether the full-text article should be sought. When there were disagreements between CF and EZ in selecting papers, the third author (L.L.) would assess the articles and make the final decision with CF and EZ. A four-phase flow diagram according to Systematic Reviews (http://www.prisma-statement.org/) was shown in Figure 1. We have used the Newcastle-Ottawa Scale (NOS), suggested by Cochrane Collaboration, for assessing the quality of each included study in the present meta-analysis. The following information was obtained from each publication: first author’s name, publication year, country origin, ethnicity, case characteristics, total number of cases and controls, numbers of each group with TGFA Taq I genotypes, respectively.

### Statistical methods

The odds ratios (ORs) and their 95% confidence intervals (95% CIs) were calculated to assess the association between TGFA Taq I polymorphism and CL/P. Pooled ORs were obtained from combination of single study by heterozygote comparison (C1C2 vs. C1C1), homozygote comparison (C2C2 vs C1C1), dominant model (C1C2 + C2C2 vs C1C1), recessive model (C2C2 vs C1C2 + C1C1) and allelic model (C2 vs C1), respectively. These comparisons were used to provide more information about the relationship between the polymorphism and the disease, assess the association in different point of view and validate the association by many ways, as well as offer the data for further study on the gene expression. Meta-analyses were performed on the total data set and separately for the source of control and disease type. We investigated the between-study heterogeneity by the Cochran’s Q test and quantified by I2. To obtain summary statistics for ORs of the polymorphism and disease, we performed initial analyses with a fixed-effect model and confirmatory analyses with a random-effect model if there was significant heterogeneity. If there is no heterogeneity, the fixed and random effects models produce similar results, and, if not, the random-effect model usually produces wider CIs than the fixed-effect model. If the P value is >0.05 of the Q test, the summary OR estimate of each study was calculated by the fixed-effect model. Otherwise, the random-effect model was used.

We assessed potential publication bias by examining funnel plots and using Egger’s test [36,37]. A funnel plot is a graph designed to check for the existence of publication bias in systematic reviews and meta-analyses. In the absence of publication bias, it assumes that the largest studies will be plotted near the average, and smaller studies will be spread evenly on both sides of the average, creating a roughly funnel-shaped distribution. Deviation from this shape can indicate publication bias. This approach is very simple to use but, sometimes, we may have doubts about the funnel asymmetry, especially if the number of studies is small. In addition, the funnel may be asymmetric due to a deficient quality of studies or because we are dealing with interventions whose effect varies with the sample of each study. For these situations, Egger’s linear regression could be used. Egger’s test plots the regression line between precision of the studies (independent variable) and the standardized effect (dependent variable). When there isn’t publication bias the regression line originates in the Y-axis zero. So much further away from zero, further evidence of publication bias. The significance of the intercept was determined by the *t*-test as suggested by Egger’s test. All of P values were two-sided and all analyses were performed using the Stata software version 11.0 (Stata Corp, College station, TX).

## Results

Detailed characteristics of each study are summarized in Table 1. A total of 20 case–control studies including 3824 cases and 7710 controls contributed to the analysis. The subjects in the study were population of Caucasian, African, Hispanic and Asian. There are some studies including more than one ethnic population. There were 14 studies containing Europeans, 4 studies including Hispanics, 3 studies involving Africans and 2 studies comprising Asians. For the Europeans, Hispanics, Africans and Asians, sample sizes ranged from 25 to 1525, 90 to 921, 17 to 69 and 12 to 100, respectively. The total sample size were 2897 cases and 6806 controls for the Europeans, 853 cases and 753 controls for the Hispanics, 41 cases and 72 controls for the Africans and 33 cases and 79 controls for the Asians. The types of controls included population-based, hospital-based and unrelated family members.

**Table 1:** Characteristics of the studies included in the meta-analysis

**Author, year**	**Country**	**Ethnicity**	**Clip type**	**Control characteristics**	**No. (case/control)**	**Case**				**Control**			
						**C1C1**	**C1C2**	**C2C2**	**C1/C2**	**C1C1**	**C1C2**	**C2C2**	**C1/C2**
BEATY [31]	USA	Caucasian	CP	HB	42/135	-	-	-	78/6	-	-	-	248/22
		Caucasian	CL/P	HB	86/135	-	-	-	163/9	-	-	-	248/22
		African	CP	HB	13/135	-	-	-	24/2	-	-	-	248/22
		African	CL/P	HB	11/135	-	-	-	22/0	-	-	-	248/22
TANABE [15]	Japan	Asian	CL/P	HB	28/73	-	-	-	49/7	-	-	-	129/17
**Lilian Jara**[21]	Chile	Hispanic	CL/P	HB	39/51	33	6	0	72/6	44	6	1	94/8
Sassani [19]	USA	Caucasian	CL/P	HB	81/84	54	26	1	134/28	70	13	1	153/15
		Asian	CL/P	HB	6/6	4	2	0	10/2	4	2	0	10/2
		African	CL/P	HB	10/7	4	5	1	13/7	4	3	0	11/3
Ardinger [12]	USA	Caucasian	CL/P	HB	78/98	59	17	2	135/21	89	8	1	186/10
Shiang [20]	USA	Caucasian	CP	HB	43/170	-	-	-	69/17	-	-	-	311/29
Hwang [22]	USA	Caucasian	CP	HB	69/284	49	20	0	118/20	239	44	1	522/46
		Caucasian	CL/P	HB	114/284	93	19	2	205/23	239	44	1	522/46
ROMITTI [30]	USA	Caucasian	CP	PB	51/295	41	10*	-	-	235	60*	-	-
		Caucasian	CL/P	PB	118/295	96	22*	-	-	235	60*	-	-
Hecht [24]	USA	Caucasian	CL/P	UFM	12/13	11	1	0	23/1	10	2	1	22/4
Chenevix [18]	Australia	Caucasian	CL/P	HB	117/113	84	30	3	198/36	94	17	2	205/21
Holder [17]	UK	Caucasian	CL/P	HB	60/60	36	14	5	86/24	55	5	0	115/5
**CHENEVIX**[29]	Australia	Caucasian	CL/P	HB	96/100	66	27	3	159/33	90	9	1	189/11
Stoll [25]	France	Caucasian	CL/P	HB	98/99	-	-	-	187/10	-	-	-	184/14
		Caucasian	CP	HB	57/99	-	-	-	104/10	-	-	-	184/14
Christensen [11]	Denmark	Caucasian	CP	PB	65/457	49	15	1	113/17	344	102	11	790/124
		Caucasian	CL/P	PB	191/457	145	45	1	335/47	344	102	11	790/124
SHAW [6]	USA	Caucasian	CP	PB	114/379	87	27*	-	-	321	58*	-	-
		Hispanic	CP	PB	35/175	34	1*	-	-	164	11*	-	-
		African	CP	PB	7/20	6	1*	-	-	18	2*	-	-
		Caucasian	CL/P	PB	245/379	212	33*	-	-	321	58*	-	-
		Hispanic	CL/P	PB	103/175	94	9*	-	-	164	11*	-	-
		African	CL/P	PB	12/20	11	1*	-	-	18	2*	-	-
Beaty [26]	USA	Caucasian	CP	HB	51/87	44	6	1	94/8	79	8	0	166/8
		Caucasian	CL	HB	26/87	21	5	0	47/5	79	8	0	166/8
		Caucasian	CL/P	HB	53/87	48	5	0	101/5	79	8	0	166/8
		African	CP	HB	12/45	10	2	0	22/2	43	2	0	88/2
		African	CL	HB	2/45	2	0	0	4/0	43	2	0	88/2
		African	CL/P	HB	10/45	9	1	0	19/1	43	2	0	88/2
Bertoja [34]	Brazil	Hispanic	CL/P	HB	140/142	114	25	1	253/27	121	21	0	263/21
PASSOS-BUENO [32]	Brazil	Hispanic	CL/P	HB	536/385	484	51	1	1019/53	344	41	0	729/41
Lidral [28]	USA	Caucasian	CL/P	PB	182/251	-	-	-	327/37	-	-	-	449/53
		Caucasian	CP	PB	62/251	-	-	-	109/15	-	-	-	449/53
Lidral [27]	USA	Caucasian	CL/P	PB	652/776	-	-	-	1204/100	-	-	-	1436/116
		Caucasian	CP	PB	97/776	-	-	-	176/18	-	-	-	1436/116

*CL*: clip lip, *CL/P*: clip lip and palate, *CP*: clip palate, *PB*: population-based control group, *HB*: hospital-based control group, *UFM*: unrelated family members control group.

C1C1, C1C2, C2C2: genotype, C1/C2: allele frequency.

*The sum of C1C2 and C2C2.

NOS results suggested that all of the included studies are high level quality with the score >6.

### Association between the genotypes of TGFA Taq1 and CL/P risk

**Figure 2:**
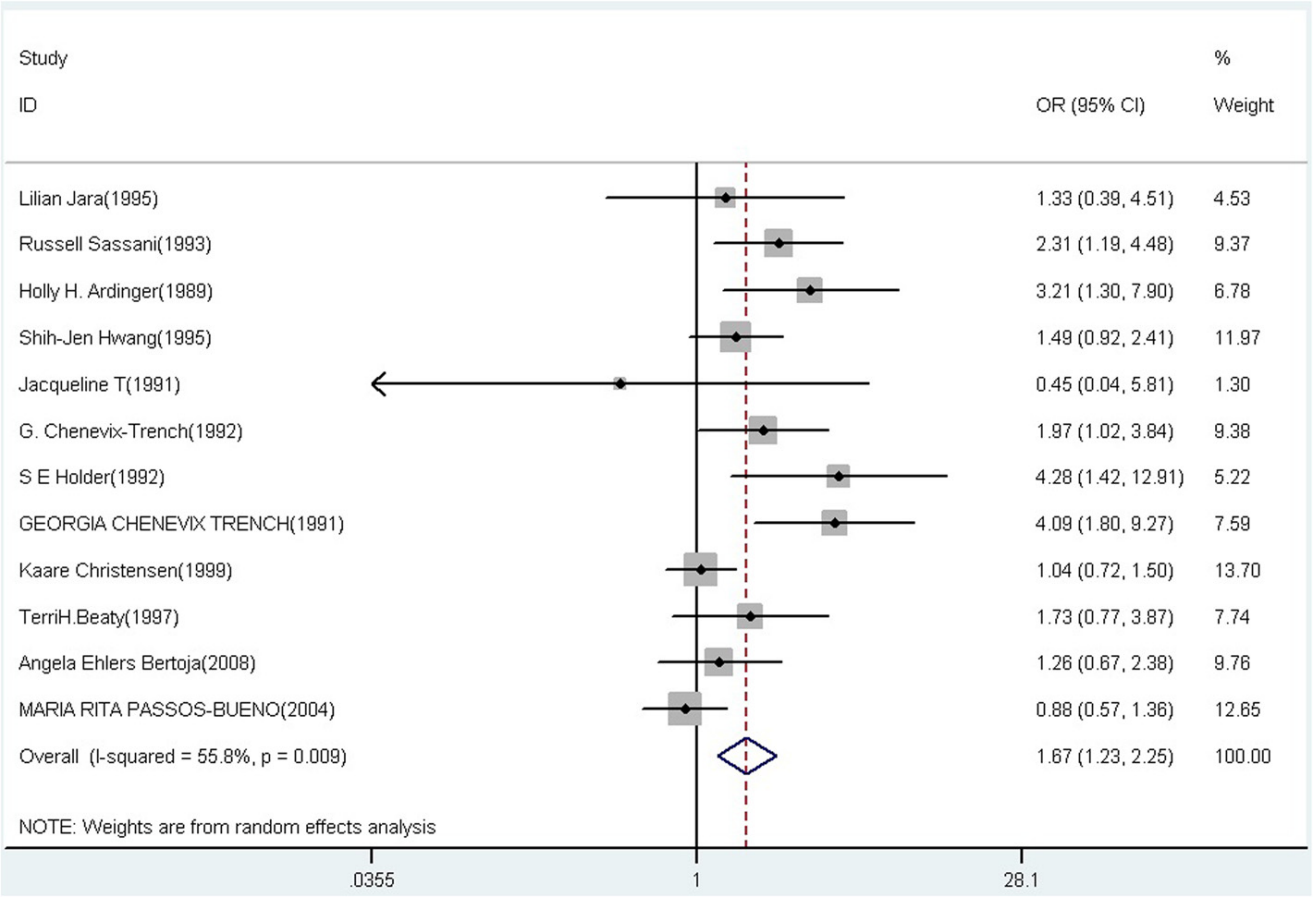
Forest plot of cancer risk associated with TGFA Taq I polymorphism under the heterozygote comparison model (C1C2 versus C1C1).

**Figure 3:**
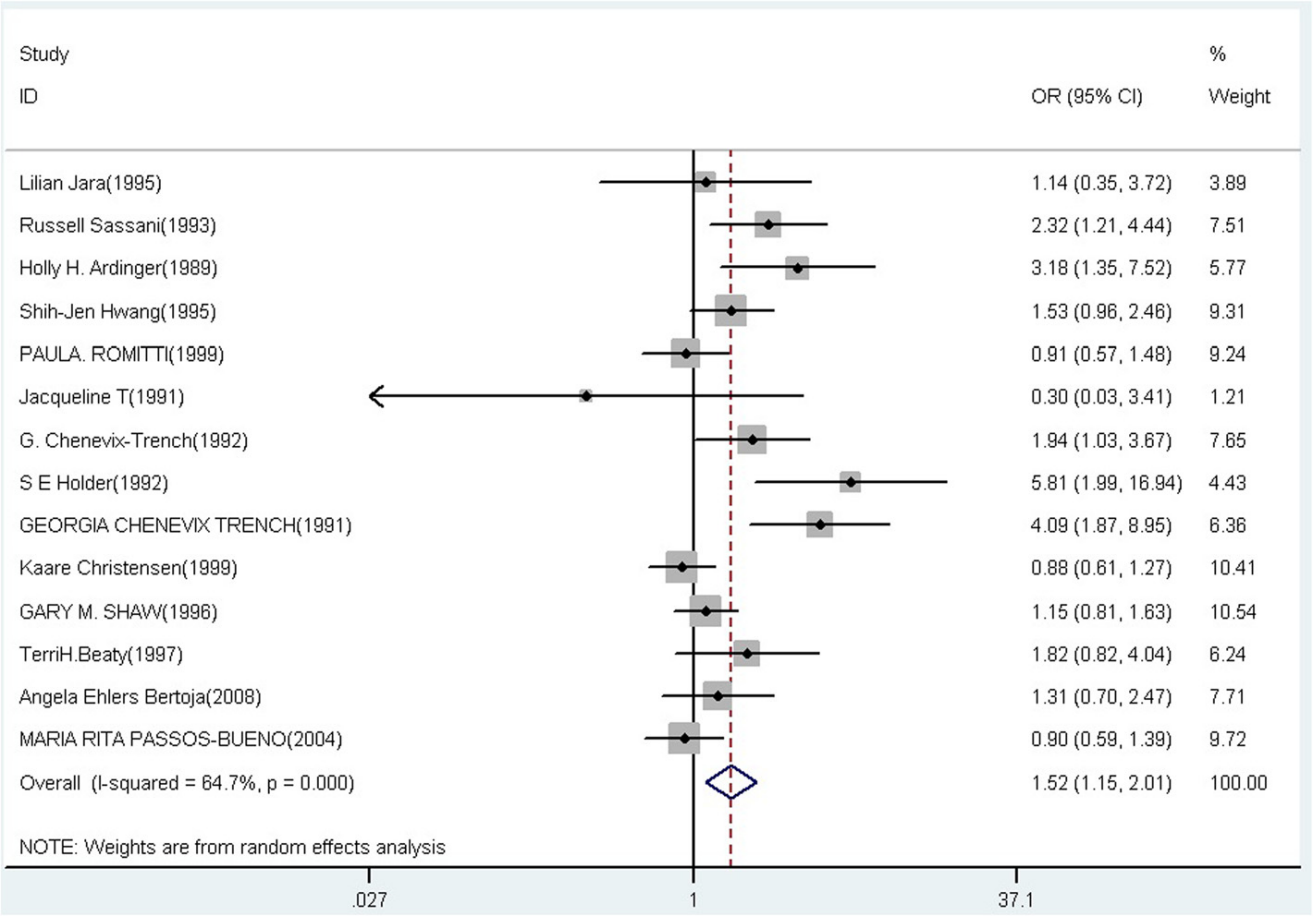
Forest plot of cancer risk associated with TGFA Taq I polymorphism under the dominant model (C1C2 + C2C2 versus C1C1).

**Figure 4:**
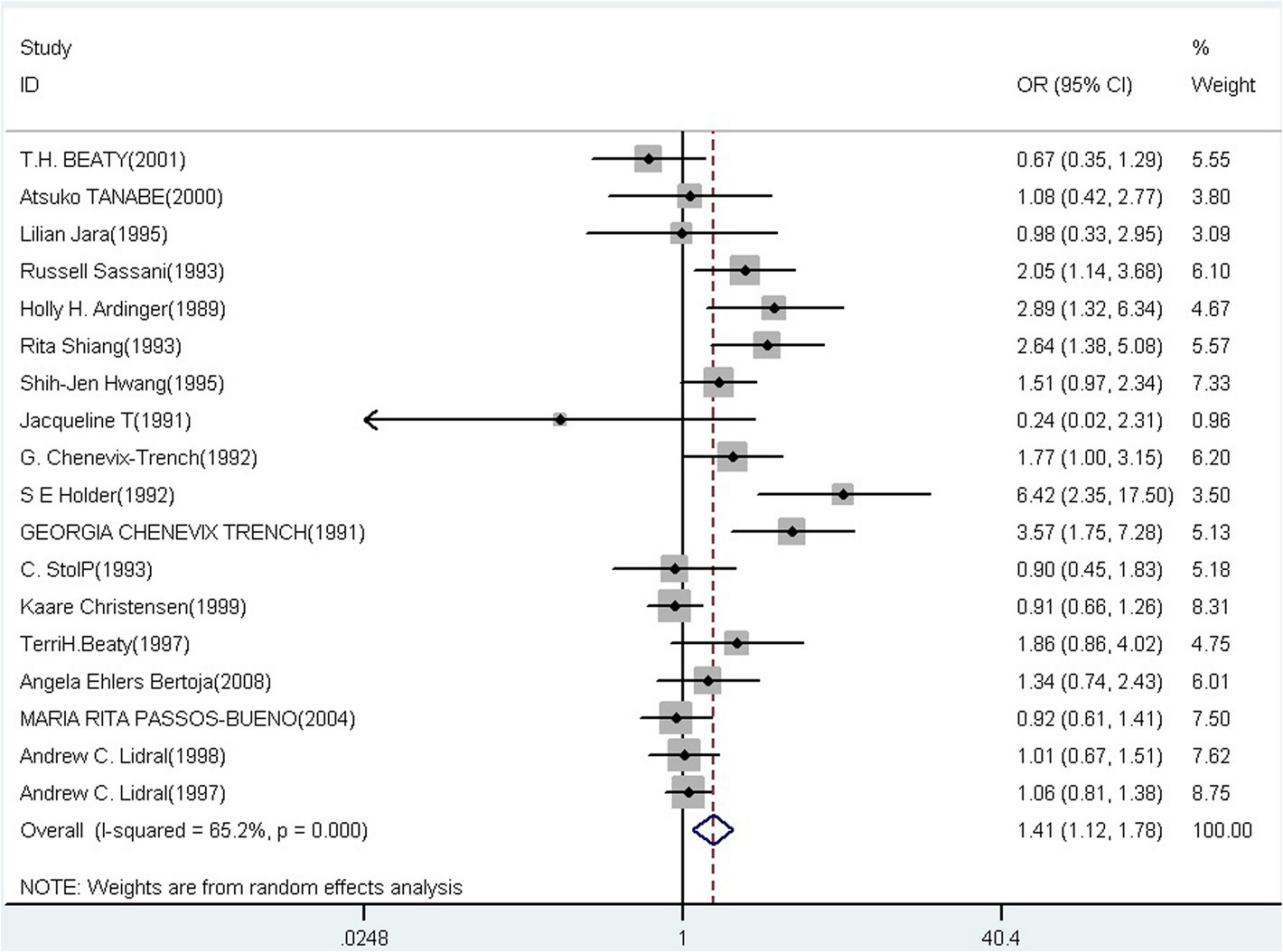
Forest plot of cancer risk associated with TGFA Taq I polymorphism under the allelic model (C2 versus C1).

A summary of the meta-analysis findings of the association between TGFA Taq I and CL/P risk is provided in Table 2. Meta-analysis showed statistically significant association between TGFA Taq I polymorphism and CL/P risk in heterozygote comparison, dominant and allelic model (C1C2 vs C1C1: OR = 1.67, 95% CI = 1.23-2.25, P = 0.009 for heterogeneity, I^2^ = 55.8%; C2C2 + C1C2 vs C1C1: OR = 1.52, 95% CI = 1.15-2.01, P < 0.001 for heterogeneity, I^2^ = 64.7%; C2 vs C1: OR = 1.41, 95% CI = 1.12-1.78, P < 0.001 for heterogeneity, I^2^ = 65.2%), but not in the homozygote and recessive model (C2C2 vs C1C1: OR = 1.57, 95% CI = 0.87-2.83, P = 0.525 for heterogeneity, I^2^ = 0.0%; C2C2 vs C1C2 + C1C1: OR = 1.43, 95% CI = 0.79-2.59, P = 0.634 for heterogeneity, I^2^ = 0.0%). Meta-analysis results of the association between TGFA Taq I polymorphism and CL/P risk under the heterozygote comparison model (C1C2 versus C1C1), the dominant model (C1C2 + C2C2 versus C1C1), and the allelic model (C2 versus C1) were also shown in Figure 2, Figure 3 and Figure 4, respectively.

**Table 2:** Association between TGFA Taq1 polymorphism and CL/P risk

**Model**	**Number of studies**	**Fixed effect**	**Random effect**	**Phet**	**I-squared (%)**
C1C2 vs. C1C1	12	1.46 [1.22,1.75]	1.67 [1.23,2.25]	0.009	55.8
C2C2 vs. C1C1	12	1.57 [0.87,2.83]	1.56 [0.78,3.16]	0.525	0.0
C1C2 + C2C2 vs. C1C1	14	1.33 [1.14,1.54]	1.52 [1.15,2.01]	0.000	64.7
C2C2 vs. C1C2 + C1C1	12	1.43 [0.79,2.59]	1.42 [0.70,2.85]	0.634	0.0
C2 vs. C1	18	1.26 [1.12,1.43]	1.41 [1.12,1.78]	0.000	65.2

### Stratified meta-analysis

Stratified analyses were conducted by ethnicity, source of control and disease type. Pooled ORs and 95% CIs of stratified meta-analysis are showed in Table 3. In the subgroup analysis by ethnicity, significantly increased CL/P risks were found among Caucasian (C1C2 vs C1C1: OR = 1.95, 95% CI = 1.34-2.86; C2C2 + C1C2 vs C1C1: OR = 1.68, 95% CI = 1.18-2.38; C2 vs C1: OR = 1.52, 95% CI = 1.14-2.02). No significantly evaluated risk was found among African and Hispanic population in any of the genetic models.

**Table 3:** Pooled ORs and 95% CIs of stratified meta-analysis

**Subgroup**	**Genotype**	**No of studies**	**Test of association**				**Test of heterogeneity**	
			**OR (95% CI)**	**Z**	**P-value**	**Model**	**P-value**	**I**^**2 **^**(%)**
**Ethnicity**								
African								
	C1C2 + C2C2 vs. C1C1	3	1.92 [0.63,1.90]	1.14	0.253	F	0.754	0.0
	C2 vs. C1	3	1.15 [0.50,2.66]	0.33	0.741	F	0.254	26.9
Caucasian								
	C1C2 vs. C1C1	9	1.95 [1.34,2.86]*	3.48	0.001	R	0.016	57.3
	C2C2 vs. C1C1	9	1.50 [0.79,2.84]	1.25	0.211	F	0.341	11.2
	C1C2 + C2C2 vs. C1C1	9	1.68 [1.18,2.38]*	2.87	0.004	R	0.000	69.9
	C2C2 vs. C1C2 + C1C1	9	1.36 [0.71,2.58]	0.93	0.354	F	0.443	0.0
	C2 vs. C1	14	1.52 [1.14,2.02]*	2.87	0.004	R	0.000	74.8
Hispanic								
	C1C2 vs. C1C1	3	1.02 [0.72,1.43]	0.08	0.935	F	0.594	0.0
	C2C2 vs. C1C1	3	1.44 [0.27,7.67]	0.42	0.672	F	0.669	0.0
	C1C2 + C2C2 vs. C1C1	4	1.04 [0.76,1.44]	0.27	0.789	F	0.792	0.0
	C2C2 vs. C1C2 + C1C1	3	1.40 [0.26,7.46]	0.4	0.692	F	0.663	0.0
	C2 vs. C1	3	1.04 [0.75,1.44]	0.23	0.816	F	0.608	0.0
**Disease**								
CP								
	C1C2 vs. C1C1	3	1.54 [1.04,2.27]*	2.14	0.032	F	0.215	34.9
	C2C2 vs. C1C1	3	1.32 [0.35,5.00]	0.40	0.687	F	0.478	0.0
	C1C2 + C2C2 vs. C1C1	5	1.45 [1.10,1.91]	2.61	0.009	F	0.281	21.0
	C2C2 vs. C1C2 + C1C1	3	1.26 [0.33,4.78]	0.33	0.738	F	0.496	0.0
	C2 vs. C1	8	1.38 [1.10,1.73]*	2.82	0.005	F	0.226	25.4
CL/P								
	C1C2 vs. C1C1	12	1.60 [1.16,2.20]*	2.89	0.004	R	0.010	55.7
	C2C2 vs. C1C1	11	1.64 [0.88,3.04]	1.57	0.116	F	0.457	0.0
	C1C2 + C2C2 vs. C1C1	11	1.46 [1.09,1.95]*	2.55	0.011	R	0.001	62.7
	C2C2 vs. C1C2 + C1C1	11	1.45 [0.82,2.78]	1.25	0.211	F	0.545	0.0
	C2 vs. C1	17	1.29 [1.01,1.66]*	2.03	0.042	R	0.000	63.1
**Source of control**								
HB								
	C1C2 vs. C1C1	10	1.84 [1.32,2.56]*	3.58	0.000	R	0.019	54.7
	C2C2 vs. C1C1	10	2.96 [1.35,2.70]*	4.70	0.000	F	0.965	0.0
	C1C2 + C2C2 vs. C1C1	10	1.99 [1.35,2.70]*	3.66	0.000	R	0.007	60.1
	C2C2 vs. C1C2 + C1C1	10	2.38 [1.06,5.55]*	5.17	0.000	F	0.968	0.0
	C2 vs. C1	14	1.63 [1.22,2.18]*	3.28	0.001	R	0.001	63.0
PB								
	C1C2 + C2C2 vs. C1C1	3	0.99 [0.79,1.24]	0.10	0.917	F	0.549	0.0
	C2 vs. C1	3	1.00 [0.83,1.20]	0.02	0.986	F	0.778	0.0

^*^OR had statistical significance with corresponding 95% CI not including 1. F: Fixed effect model; R: Random effect model.

In the subgroup analysis according to disease type, the ORs of the heterozygote comparison, dominant and allelic model with CL/P are statistically significant (C1C2 vs C1C1: OR = 1.60, 95% CI = 1.16-2.20; C2C2 + C1C2 vs C1C1: OR = 1.46, 95% CI = 1.09-1.95; C2 vs C1: OR = 1.29, 95% CI = 1.01-1.66). For the CP, the significant results were observed in heterozygote comparison, dominant and allelic model (C1C2 vs C1C1: OR = 1.54, 95% CI = 1.04-2.27; C2C2 + C1C2 vs C1C1: OR = 1.45, 95% CI = 1.10-1.19; C2 vs C1: OR = 1.38, 95% CI = 1.10-1.73).

In the subgroup analysis by control characteristics, the ORs of the heterozygote comparison, homozygote, dominant, recessive and allelic model for the hospital-based control are statistically significant (C1C2 vs C1C1: OR = 1.84, 95% CI = 1.32-2.56; C2C2 vs C1C1: OR = 2.96, 95% CI = 1.35-2.70; C2C2 + C1C2 vs C1C1: OR = 1.99, 95% CI = 1.35-2.70; C2C2 vs C1C2 + C1C1: OR = 2.38, 95% CI = 1.06-5.55; C2 vs C1: OR = 1.63, 95% CI = 1.22-2.18). While, no statistically significant association was found in population-based controls.

Heterogeneity between studies was observed in overall comparisons and also subgroup analyses. Thus, meta-analyses were performed using random-effect models (Table 2).

**Figure 5:**
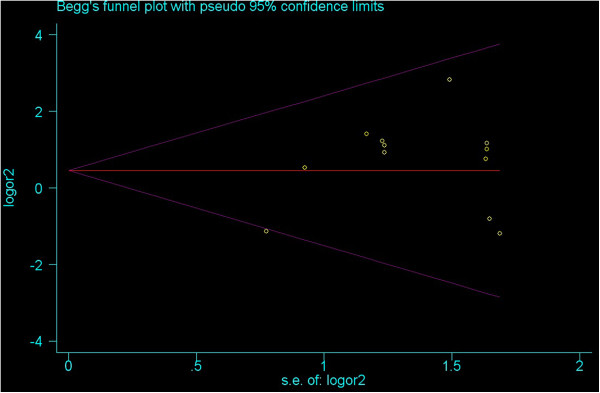
Funnel plot under the heterozygote comparison model (C1C2 versus C1C1).

**Figure 6:**
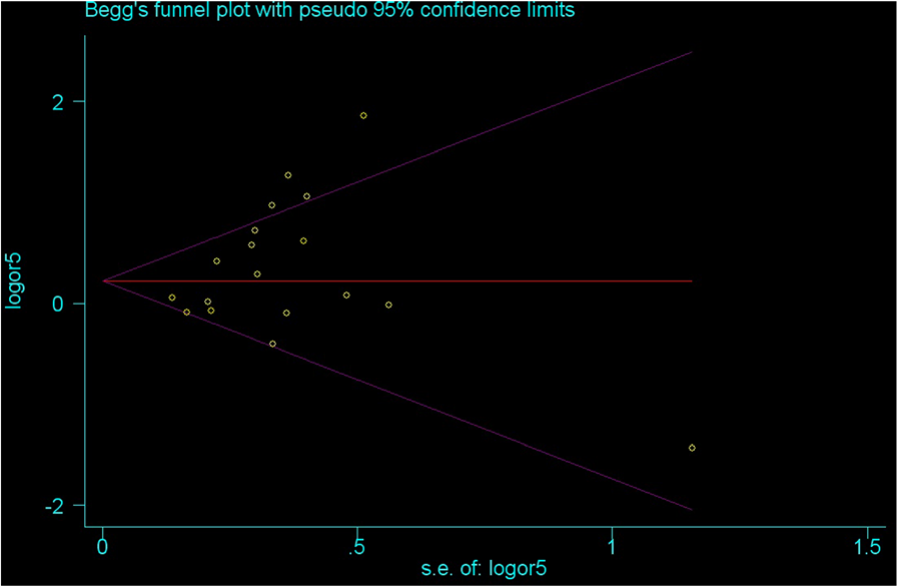
Funnel plot under the allelic model (C2 versus C1).

To explore potential sources of heterogeneity in this meta-analysis, meta-regression analyses were implemented. The covariates included ethnicity and source of control. In all of the heterozygote comparison, homozygote, dominant, recessive and allelic models, above potential factors were probably not the major sources of heterogeneity (P-values were all >0.05 or near 0.05). The heterogeneity might attribute to other factors, the insufficient data limited to identify their sources only using meta-regression.No publication bias was detected by either the inverted funnel plot or Egger’s test. The shapes of the funnel plots seemed approximately symmetrical and P values of the Egger’ tests were not statistical significant (P values were all >0.05). Figure 5 and Figure 6 showed the funnel plot under the heterozygote comparison model (C1C2 versus C1C1) and the allelic model (C2 versus C1).

## Discussion

The individual susceptibility plays an important role in the development of most diseases. Therefore, the studies of genetic susceptibility are helpful for disease prevention, diagnosis, and treatment.

TGFA is a well characterized mammalian growth factor. Previous studies found that although TGFA is expressed in mice during palatogenesis and mice with a null mutation of the TGFA gene have abnormal skin, hair, and eyes, but they do not have CL/P [38,39]. TGFA is a likely ligand for epidermal growth factor receptor and newborn epidermal growth factor receptor-negative/-negative mice have high incidence of CL/P which may explain the association between TGFA polymorphisms and CL/P [40]. In 1989, it was firstly published that the TGFA Taq I polymorphism contributed to the development CL/P in humans. From then on, a growing number of studies have been done to examine the relationship between TGFA Taq I polymorphism and the risks of CL/P. However, the results are not consistent. In order to better understand the association, we subjected previously published data to meta-analysis to evaluate genetic associations between the TGFA Taq I polymorphism and CL/P susceptibility.

Through this meta-analysis we found that TGFA Taq I polymorphism increased the risk of CL/P. Mitchell adopted a reappraisal and showed that there was a statistically significant association between TGFA and CL/P, However, there was evidence of significant heterogeneity from different studies, regarding an association between genetic variation at the TGFA locus and CL/P remains inconclusive [41]. A meta-analysis of gene-environment interaction showed the suggestive evidence for gene-environment interaction between the infant’s genotype at the Taq1 marker in TGFA and maternal smoking was limited to CP [42]. Meta-review of thyroid cancer gene expression profiling studies identifies important diagnostic biomarkers, including relatively novel or uncharacterized genes such as TGFA [43]. These results of systematic reviews in some extent support the findings in the present meta-analysis.

In order to reduce heterogeneity, we performed the stratified analyses. It is well know that the incidence of most genetic polymorphisms could vary between different ethnic populations and the wide range in TGFA Taq I allele frequencies across different studies suggests the heterogeneity between populations may exist [28]. In the subgroup analysis by ethnicity, we found that TGFA Taq I polymorphism increased the risk of CL/P in Caucasian population, which agreed with Ardinger and colleagues [12], Shiang and colleagues [20], Hwang and colleagues [22], Chenevix-Trench and colleagues [18], Holder and colleagues [17], and disagreed with Lidral and colleagues [27], [28], Stoll and colleagues [25] and Christensen and colleagues [11]. In the subgroup analysis by disease type, we found the ORs of different disease type were statistically significant, which suggested disease type was not mainly result the heterogeneity. In the subgroup analyses of control characteristics, only the ORs of hospital-based control groups were statistically significant. Therefore, subgroup analyses suggested that ethnicity and control characteristics might contribute to the heterogeneity in this meta-analysis. The meta-regression analyses suggested that ethnicity or control characteristics were probably not the major sources of heterogeneity. The heterogeneity might attribute to other factors. The insufficient data are limited to identify the source of heterogeneity only using meta-regression.

Despite trying our best to perform a comprehensive meta-analysis, some limitations exist in our study. Although the results for publication bias in our study were not statistically significant, our analysis used published international studies, which could arose publication bias. Lack of the original data of available studies limited our further evaluation of potential interactions, such as age, gender, family history, environmental factors and lifestyle. There were not significant results in stratified analyses among African and Hispanic population, because there were too few studies after stratifying. Therefore, more studies are needed to provide more evidence on the association between TGFA polymorphism and CL/P in different ethnic populations.

Although CL/P is a complex disease, our study provided evidence showing the important role of TGFA genes polymorphisms in the development of CL/P, genetic factors determining disease susceptibility and severity may facilitate personalized medicine. Further understanding of the interactions between genetic regulatory mechanisms is critical for discovering new therapies for managing human CL/P. Specifically, targeted therapy about the polymorphisms of related genes might be a promising avenue for future CL/P diagnosis and treatment.

In summary, our meta-analysis supports that the TGFA Taq I polymorphism is more likely to contribute to the susceptibility of CL/P, especially in the subgroup of Caucasian population.

## Conclusion

TGFA Taq I polymorphism may be associated with the risk of CL/P.

## Competing interests

The authors declare that they have no competing interests.

## Authors’ contributions

CF participated in selecting the study, extracting the data, performing the statistical analysis and drafting the manuscript. EZ and WD participated in study selection, data extraction and manuscript drafting. ZX collected and extracted the data. YZ and LL participated in study selection and the statistical analysis. All authors read and approved the final manuscript.

## Pre-publication history

The pre-publication history for this paper can be accessed here:

http://www.biomedcentral.com/1472-6831/14/88/prepub

## References

[B1] Melnick M Jaskoll T Marazita M Localization of H-2^K^ in developing mouse palates using monoclonal antibody J Embryol Exp Morphol 1982 70 45 60 6754848

[B2] Wyszynski DF Beaty TH Review of the role of potential teratogens in the origin of human nonsyndromic oral clefts Teratology 1996 53 309 317 8879089 10.1002/(SICI)1096-9926(199605)53:5<309::AID-TERA5>3.0.CO;2-W

[B3] Czeizel A Nagy E A recent aetiological study on facial clefting in Hungary Acta Pediatr Hung 1986 27 145 166 3756014

[B4] Khoury MJ Gomez-Farias M Mulinare J Does maternal cigarette smoking during pregnancy cause cleft lip and palate in offspring? Am J Dis Child 1989 143 333 337 2644816 10.1001/archpedi.1989.02150150091023

[B5] Werler MM Lammer EJ Rosenberg L Mitchell AA Maternal cigarette smoking during pregnancy in relation to oral clefts Am J Epidemiol 1990 132 926 932 2239907 10.1093/oxfordjournals.aje.a115735

[B6] Shaw GM Wasserman CR Lammer EJ O’Malley CD Murray JC Basart AM Tolarova MM Orofacial clefts, parental cigarette smoking, and transforming growth factor-alpha gene variants Am J Hum Genet 1996 58 551 561 8644715 PMC1914570

[B7] Tolarova MM Orofacial clefts in Czechoslovakia: incidence, genetics and prevention of cleft lip and palate over a 19-year period Scand J Plast Reconstr Surg 1987 21 19 25 10.3109/02844318709083574 3296142

[B8] Tolarova MM Harris JA Reduced recurrence of orofacial clefts after periconceptional supplementation with high-dose folic acid and multivitamins Teratology 1995 51 71 78 7660324 10.1002/tera.1420510205

[B9] Hayes C Werler MM Willett WC Mitchell AA Case–control study of periconceptional folic acid supplementation and oral clefts Am J Epidemiol 1996 143 1229 1234 8651221 10.1093/oxfordjournals.aje.a008710

[B10] Munger RG Romitti PA Daack-Hirsch S Burns TL Murray JC Hanson J Maternal alcohol use and risk of orofacial cleft birth defects Teratology 1996 54 27 33 8916367 10.1002/(SICI)1096-9926(199607)54:1<27::AID-TERA4>3.0.CO;2-0

[B11] Christensen K Olsen J Nørgaard-Pedersen B Basso O Støvring H Milhollin-Johnson L Murray JC Oral clefts, transforming growth factor alpha gene variants, and maternal smoking: a population-based case–control study in Denmark, 1991–1994 Am J Epidemiol 1999 149 248 255 9927220 10.1093/oxfordjournals.aje.a009799

[B12] Ardinger HH Buetow KH Bell GI Bardach J Van-Demark DR Murray JC Association of genetic variation of the transforming growth factor-alpha gene with cleft lip and palate Am J Hum Genet 1989 45 348 353 2570526 PMC1683414

[B13] Dixon MJ Garner J Fergusson MW Immunolocalization of epidermal growth factor (EGF), EGF receptor and transforming growth factor alpha (TGFa) during murine palatogenesis *in vivo* and *in vitro* Anat Embryol 1991 184 83 91 1928747 10.1007/BF01744264

[B14] Lee DC Rochford R Todaro GJ Villarreal LP Developmental expression of rat transforming growth factor-*a* mRNA Mol Cell Biol 1985 5 3644 3646 3870134 10.1128/mcb.5.12.3644-3646.1985 PMC369199

[B15] Tanabe A Taketani S Endo-Ichikawa Y Tokunaga R Ogawa Y Hiramoto M Analysis of the candidate genes responsible for nonsyndromic cleft lip and palate in Japanese people Clin Sci 2000 99 105 111 10918043

[B16] Vieira AR Association between the transforming growth factor alpha gene and nonsyndromic oral clefts: a HuGE review Am J Epidemiol 2006 163 790 810 16495466 10.1093/aje/kwj103

[B17] Holder SE Vintiner GM Farren B Malcolm S Winter RM Confirmation of an association between RFLPs at the transforming growth factor-alpha locus and non-syndromic cleft lip and palate J Med Genet 1992 29 390 392 1352354 10.1136/jmg.29.6.390 PMC1015988

[B18] Chenevix-Trench G Jones K Green AC Duffy DL Martin NG Cleft lip with or without cleft palate: associations with transforming growth factor alpha and retinoic acid receptor loci Am J Hum Genet 1992 51 1377 1385 1361101 PMC1682912

[B19] Sassani R Bartlett SP Feng H Goldner-Sauve A Haq AK Buetow KH Gasser DL Association between alleles of the transforming growth factor-alpha locus and the occurrence of cleft lip Am J Hum Genet 1993 45 565 569 10.1002/ajmg.1320450508 8096116

[B20] Shiang R Lidral AC Ardinger HH Buetow KH Romitti PA Munger RG Murray JC Association of transforming growth-factor alpha gene polymorphisms with nonsyndromic cleft palate only (CPO) Am J Hum Genet 1993 53 836 843 8105683 PMC1682388

[B21] Jara L Blanco R Chiffelle I Palomino H Carreño H Association between alleles of the transforming growth factor alpha locus and cleft lip and palate in the Chilean population Am J Hum Genet 1995 57 548 551 10.1002/ajmg.1320570406 7573126

[B22] Hwang SJ Beaty TH Panny SR Street NA Joseph JM Gordon S McIntosh I Francomano CA Association study of transforming growth factor alpha (TGF alpha) TaqI polymorphism and oral clefts: indication of gene-environment interaction in a population-based sample of infants with birth defects Am J Epidemiol 1995 141 629 636 7702037 10.1093/oxfordjournals.aje.a117478

[B23] Shaw GM Wasserman CR Murray JC Lammer EJ Infant TGF-alpha genotype, orofacial clefts, and maternal periconceptional multivitamin Use Cleft Palate Craniofcial J 1998 35 366 370 10.1597/1545-1569_1998_035_0366_itagoc_2.3.co_2 9684776

[B24] Hecht JT Wang YP Blanton SH Michels VV Daiger SP Cleft lip and palate: no evidence of linkage to transforming growth factor alpha Am J Hum Genet 1991 49 682 686 1679292 PMC1683150

[B25] Stoll C Qian JF Feingold J Sauvage P May E Genetic variation in transforming growth factor alpha: possible association of Bam HI polymorphism with bilateral sporadic cleft lip and palate Hum Genet 1993 92 81 82 8103504 10.1007/BF00216150

[B26] Beaty TH Maestri NE Hetmanski JB Wyszynski DF Vanderkolk CA Simpson JC Testing for interaction between materrnal somking and TGFA genotype among oral cleft case born in Maryland 1992–1996 Cleft Palate Craniofcial J 1997 34 447 454 10.1597/1545-1569_1997_034_0447_tfibms_2.3.co_2 9345615

[B27] Lidral AC Murray JC Buetow KH Basart AM Schearer H Shiang R Naval A Layda E Magee K Magee W Studies of the candidate genes TGFB2, MSX1, TGFA and TGFB3 in the etiology of cleft lip and palate in the Philippines Cleft Palate Craniofac J 1997 34 1 6 9003904 10.1597/1545-1569_1997_034_0001_sotcgt_2.3.co_2

[B28] Lidral AC Romitti PA Basart AM Doetschman T Leysens NJ Daack-Hirsch S Semina EV Johnson LR Machida J Burds A Parnell TJ Rubenstein JL Murray JC Association of MSX1 and TGFB3 with nonsyndromic clefting in humans Am J Hum Genet 1998 63 557 568 9683588 10.1086/301956 PMC1377298

[B29] Chenevix-Trench G Jones K Green A Martin N **Further Evidence for an Association Between Genetic Variation in Transforming Growth Factor Alpha and Cleft Lip and Palate** Am J Hum Genet 1991 48 1012 1013 1673285 PMC1683066

[B30] Romitti PA Lidral AC Munger RG Daack-Hirsch S Burns TL Murray JC Candidate genes for nonsyndromic cleft lip and palate and maternal cigarette smoking and alcohol consumption: evaluation of genotype-environment interactions from a population-based case–control study of orofacial clefts Teratology 1999 59 39 50 9988882 10.1002/(SICI)1096-9926(199901)59:1<39::AID-TERA9>3.0.CO;2-7

[B31] Beaty TH Wang H Hetmanski JB Fan YT Zeiger JS Liang KY Chiu YF Vanderkolk CA Seifert KC Wulfsberg EA Raymond G Panny SR McIntosh I A case–control study of nonsyndromic oral clefts in Maryland Ann Epidemiol 2001 11 434 442 11454503 10.1016/s1047-2797(01)00222-8

[B32] Passos-Bueno MR Gaspar DA Kamiya T Tescarollo G Rabanéa D Richieri-Costa A Alonso N Araújo B Transforming growth factor-ɑ and nonsyndromic cleft lip with or without palate in Brazilian patients: results of a large case–control study Cleft Palate Craniofcial J 2004 41 387 391 10.1597/03-054.1 15222785

[B33] Chevrier C Bahuau M Perret C Iovannisci DM Nelva A Herman C Vazquez MP Francannet C Robert-Gnansia E Lammer EJ Cordier S Genetic susceptibilities in the association between maternal exposure to tobacco smoke and the risk of nonsyndromic oral cleft Am J Med Genet A 2008 146A 2396 2406 18698632 10.1002/ajmg.a.32505

[B34] Ehlers Bertoja A Sampaio Alho C De Franca E Menegotto B Miriam Robinson W TGFA/TAQ I polymorphism in nonsyndromic cleft lip and palate patients from Rio Grande Do Sul, Brazil Cleft Palate Craniofcial J 2008 45 539 544 10.1597/07-087.1 18788876

[B35] Zhu J Hao L Li S Bailey LB Tian Y Li Z MTHFR, TGFB3, and TGFA polymorphisms and their association with the risk of non-syndromic cleft lip and cleft palate in China Am J Med Genet A 2010 152A 291 298 20082468 10.1002/ajmg.a.33113

[B36] Begg CB Mazumdar M Operating characteristics of a rank correlation test for publication bias Biometrics 1994 50 1088 1101 7786990

[B37] Egger M Davey Smith G Schneider M Minder C Bias in meta-analysis detected by a simple, graphical test BMJ 1997 315 629 634 9310563 10.1136/bmj.315.7109.629 PMC2127453

[B38] Mann GB Fowler KJ Gabriel A Nice EC Williams RL Dunn AR Mice with a null mutation of the TGFa gene have abnormal skin architecture, wavy hair, and curly whiskers and often develop corneal inflammation Cell 1993 73 249 261 8477444 10.1016/0092-8674(93)90227-h

[B39] Luetteke NC Qiu TH Peiffer RL Oliver P Smithies O Lee DC TGFa deficiency results in hair follicle and eye abnormalities in targeted and waved-1 mice Cell 1993 73 263 278 8477445 10.1016/0092-8674(93)90228-i

[B40] Miettinen PJ Chin JR Shum L Slavkin HC Shuler CF Derynck R Werb Z Epidermal growth factor receptor function is necessary for normal craniofacial development and palate closure Nat Genet 1999 22 69 73 10319864 10.1038/8773

[B41] Mitchell LE Transforming growth factor alpha locus and nonsyndromic cleft lip with or without cleft palate: a reappraisal Genet Epidemiol 1997 14 231 240 9181353 10.1002/(SICI)1098-2272(1997)14:3<231::AID-GEPI2>3.0.CO;2-8

[B42] Zeiger JS Beaty TH Liang KY Oral clefts, maternal smoking, and TGFA: a meta-analysis of gene-environment interaction Cleft Palate Craniofac J 2005 42 58 63 15643916 10.1597/02-128.1

[B43] Griffith OL Melck A Jones SJ Wiseman SM Meta-analysis and meta-review of thyroid cancer gene expression profiling studies identifies important diagnostic biomarkers J Clin Oncol 2006 24 5043 5051 17075124 10.1200/JCO.2006.06.7330

